# Greenium of green securitization: Does external certification matter?

**DOI:** 10.1371/journal.pone.0306814

**Published:** 2024-08-12

**Authors:** Xiru Li, Bo Zhu, Yufei Zhang

**Affiliations:** School of Finance and Institute of Chinese Financial Studies, Southwestern University of Finance and Economics, Chengdu, China; University of Rome Tor Vergata: Universita degli Studi di Roma Tor Vergata, ITALY

## Abstract

The financing costs of green asset-backed securities (ABS) are deeply affected by the increased information asymmetry and greenwashing risk resulting from risk transferring in securitization. To attract potential investors, many ABS issuers obtain external certifications, yet it is unclear whether they pay off financially. Based on a sample of 588 green ABS issued in China for 2016–2022, this paper examines the impact of external certification in the form of green certification and reputation of financial intermediaries involved in the issuance on the yield discount of green ABS over the paired non-green ABS. The empirical findings show that both external certifications lower the greenium of green ABS by serving as favorable signals and mitigating greenwashing concerns, especially in non-financial industry and the securities exchange market. Moreover, the information asymmetry and credit risk of issuers enhance the pricing effect of financial intermediary certification but undermine that of green certification. Our findings provide valuable implications to facilitate the financing efficiency of green financial markets and promote global low-carbon transition.

## 1. Introduction

Sustainable energy projects require substantial investments that exceed the capacity of traditional financing methods, especially during the transition to low-carbon energy sources [1]. To bridge this gap, various green financial products have been developed to support environmentally friendly projects. Among these, green asset-backed securities (ABS) show greater growth potential than green loans and bonds, which are limited in scale, particularly in developing countries with overstretched government finances. ABS fund projects by packaging and selling underlying illiquid assets (e.g., wind farms’ on-grid electricity fee income rights) [2]. These securities are further viewed as green if the proceeds are invested in green projects or if underlying assets are eco-friendly. Green ABS are then particularly appealing for traditional energy companies with high leverage and new energy companies with low debt capacity. Nevertheless, green securitization transfers project risks from issuers to investors, which can lead to lax screening and increased moral hazard [3]. This raises concerns about greenwashing and reduces investors’ willingness to pay a greenium—the difference between at-issue yield spreads on green ABS and the similar conventional ABS. To mitigate these issues, issuers seek possible methods to convey information about their product quality to investors. One such method, which we investigate, is the external certification provided by third parties.

Despite the noteworthy potential of the green ABS market, concerns about greenwashing and the damaged reputation of asset-backed securitization following the global financial crisis have impeded its growth [4–6]. Green ABS issuances are characterized by heightened information asymmetry between inside managers and outside investors due to complex bond structures, uninformative investors [7], and inadequate information disclosure. Consequently, the environmental promises of green ABS issuers are more likely to be questioned. External certification, however, may be an effective tool to alleviate this issue by lowering information asymmetry [4, 8, 9]. For example, firms can deliver favorable signals regarding their quality to market participants by obtaining a third-party green certificate or a business relationship with reputable financial intermediaries, resulting to reduced adverse selection costs. Or moral hazard can be mitigated due to certifiers’ monitoring during the post-issuance period. On the other hand, the effectiveness of certification may be undermined by collusion between issuers and third parties or insufficient ex-post supervision by certifiers [3, 10, 11]. Owing to these mixed conclusions, we investigate the role of external certification in green ABS market where information asymmetry on greenness is relatively severe.

Furthermore, we narrow our focus to two types of certifications: green certification and financial intermediary certification, which are particularly relevant to green ABS issuance and are optional for potential issuers. On the one hand, several studies have demonstrated the importance of green certificates issued by professional third-party agencies in green financial market [4, 12]. These certificates are awarded following a comprehensive evaluation of the environmental benefits of the underlying project and the use of funds raised, explicitly signifying the level of product greenness [13]. On the other hand, the quality of green ABS is implicitly certified based on the certifiers’ reputation [14]. That is, the business relationships with well-known financial intermediaries [15–19] may also reveal the issuers’ private information. However, few papers have explored the differential roles of both certification tools in generating a greenium, let alone how the characteristics of them and issuers correlate with the certification effects.

**Fig 1:**
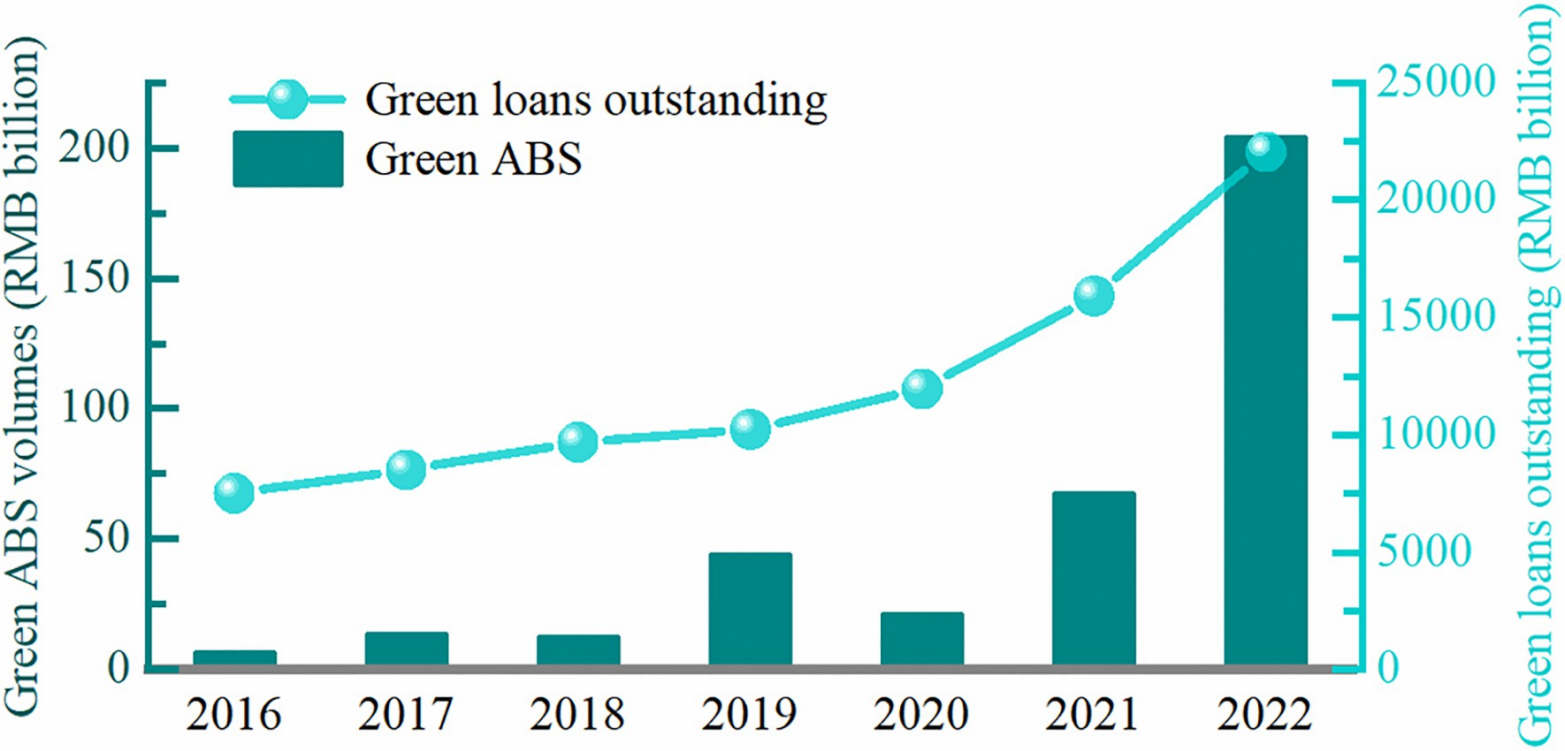
Chinese green loans outstanding and green ABS issuance volume. The issuance volume of Chinese green ABS is on the left-hand axis. The outstanding green loan volume is on the right-hand axis. The data are obtained from the Wind database.

This study uses the Chinese green ABS market as a sample to examine the aforementioned issues for several reasons. Firstly, the Chinese government is a global pioneer in building a green financial system. As depicted in **Fig 1**, the issuance volume of green ABS increased from RMB 6.13 billion in 2016 to RMB 203.56 billion in 2022, with average annual growth of 79%. That is, the Chinese green ABS market is booming, presenting an ideal setting to explore our research questions. Secondly, relatively immature environmental information disclosure systems and limited awareness of disclosure practices lead to more pronounced information asymmetry in emerging financial markets compared to developed markets [20–22]. As a result, the certification mechanism can differ substantially between them. Thirdly, obtaining large-scale and affordable green financing poses a significant challenge, particularly for underdeveloped economies like China, where the total issue size of Chinese green ABS lags behind that of green loan (Fig 1). Thus, our study might provide a valuable implication for expanding the green ABS market, especially for emerging countries committed to emission reduction targets.

Using green ABS issued by Chinese firms from 2016 to 2022, this paper examines the impact of external certification on the greenium of green ABS and further analyzes the influential mechanism from both ex-ante and ex-post perspectives. We find that both green and intermediary certifications reduce the greenium by ex-ante signaling channel rather than ex-post incentive channel. That is, both certifications mitigate investors’ greenwashing concerns ex ante the offering and lower the adverse selection costs. Additionally, when we examine the moderating effect of the information environment and issuers’ credit quality, significant differences emerge between green and financial intermediary certification. In contrast to the latter, the former loses its significance if issuers lack transparency or have low credit quality. This suggests that the credibility of green certificates may be questioned under unfavorable conditions, especially in immature and less regulated green certification markets. Finally, we conduct several heterogeneity tests to gain deeper insights into the certification effect. The results indicate that the effect is particularly pronounced in subsamples of non-financial firms and the security exchange market.

This paper makes several important contributions to the existing literature. Firstly, our analysis is related to the literature on greenwashing risk and greenium [4, 12]. However, these studies mostly focus on green bond samples. Our analysis complements their findings by offering evidence from the green ABS market, characterized by more complicated product structures and higher moral hazard and adverse selection costs. Moreover, this paper contributes to the recent debate on the pricing effects of observable certification under asymmetric information [11, 14, 17]. Previous works on external certification have adopted measures that reflect information generated by third-party organizations but do not further differentiate the effects of different certificates. We identify two types of external certifications and reveal their respective influencing mechanisms. Finally, our findings offer specific implications for firms seeking suitable certification tools. Although the positive role of external certification in accessing external finance has been confirmed in existing literature [23], the potential channels and heterogeneous effects have not been fully explored. This study provides valuable insights for expanding the green ABS market and promoting low-carbon energy transition.

The remainder of this study is organized as follows. Section 2 reviews relevant literature on green ABS and external certification and develops the research hypotheses. Section 3 introduces the source of data and the empirical methods. The empirical findings and robustness tests are presented in Section 4. Section 5 concludes this paper with some recommendations and presents limitations and possible extensions.

## 2. Literature review and hypothesis development

### 2.1 Green ABS

A growing body of literature has recently examined green finance, driven by the global transition to a low-carbon economy [24, 25]. Green finance is defined as “financing of investments that provide environmental benefits” and encompasses a wide variety of financial instruments that support climate-friendly projects. The popularity of these financial instruments has grown due to both reputational and financial considerations [26, 27]. As an essential component of green finance, green securitization frees up capital for new green projects. Specifically, green ABS can be distinguished from conventional ABS in two key aspects according to alternative definitions [28]. On one hand, greenness is reflected in the use of proceeds from issuing ABS. Green ABS issuers raise capital by packaging and selling fundamental assets, then investing the proceeds in eco-friendly projects. In this context, the underlying assets can be brown or even stranded assets. This approach allows issuers to raise funds for the low carbon transition while transferring climate-related risk exposure to investors. On the other hand, a securitisation can also be treated as green if the packaged cash flow is generated from environmentally friendly projects. Therefore, this innovative financing channel undoubtedly fills the financing gap in the low-carbon transition.

However, despite the multiplier effect of green ABS in scaling up green investments [28], their issuance events may be opaquer than those of other green financial tools. Specifically, green ABS originators can transfer the risk exposure, such as credit risk or climate risk, of the underlying asset by establishing Special Purpose Vehicles (SPVs), thus safeguarding other assets [29, 30]. Although information asymmetry between issuers and investors is common in financial market, this mechanism can exacerbate ex-post moral hazard and ex-ante adverse selection problems in ABS markets compared to corporate bond markets [3, 7, 31, 32]. Consequently, it is difficult to price green ABS rationally.

Investor concerns about greenwashing are intensified due to the significant information asymmetry associated with green ABS offerings. Greenwashing refers to the practice of making unsubstantiated or misleading claims about environmental commitment [12]. Firms may engage in greenwashing due to investor demand, market pressure, environmental regulation, or its impact on the cost of capital [33–35]. Thus, the information asymmetry regarding the green attributes of green ABS projects leads to investors’ skepticism about their true environmental impact [33, 36]. In particular, the greenness of the fundamental assets and use of proceeds are often unobservable. This skepticism and perceived risk increase funding costs [37], specifically reflected in the greenium within the offering yield spreads [11]. While many studies have examined the existence of a greenium using green bond market data, no consensus has been reached yet [11, 12, 38–42], and empirical evidence from green ABS markets remains limited.

Because the green ABS issuances are typically private placement offerings and some related data are not publicly available, the green ABS market has received relatively less attention in research. Some studies employ qualitative methodologies to analyze the current status and characteristics of the green ABS market, providing valuable implications for its future development [43, 44]. From a quantitative viewpoint, researchers examine the impact of green ABS issuances on energy efficiency or investigate the potential determinants of green ABS offering prices [28, 45, 46]. These determinants include the size, maturity, and rating of the security, the greenness and default probability of the underlying assets, as well as the correlations between individual assets in the asset pool. Although the information environment plays a crucial role in asset pricing [47], there is a notable gap in empirical studies that specifically focus on the impact of external reviews on the greenium of green ABS from the perspective of information asymmetry.

### 2.2 External certification and greenium of green ABS

The external certification may affect the greenium of green ABS through both the “signaling channel” and the “incentive channel”. The signaling channel operates based on signaling theory, which suggests that information transmission can reduce information asymmetry in external financing markets if the signals are voluntary and difficult to imitate. The green certificate obviously satisfies these two requirements. Currently, third-party green certification is encouraged but not mandatory for green ABS in China. Besides, firms who pursue greenwashing strategies would be less inclined to obtain green certificates since their greenwashing behavior may be detected by certifiers and then the green ABS issue may fail. Financial intermediary certification, although not in the form of explicit labels, can still implicitly guarantee product quality through intermediary reputation [18, 48]. First, firms have multiple options when selecting financial intermediaries, which satisfies the first requirement of being an effective signal. In addition, reputable intermediaries have more to lose in terms of reputational capital if product defaults occur, thereby motivating them to be more selective in their service provision [8, 49]. Therefore, both external certifications may reduce adverse selection costs stemming from information asymmetry on greenness, and then improve efficiency in the green ABS market.

External certification may also affect the greenium of green ABS through the incentive effect. Since the behaviors of green ABS managers cannot be fully observed, they may prioritize maximizing their private benefits over those of investors in the post-issuance period [50]. For example, they might invest the proceeds from green ABS in brown projects with higher profits and lower risks than green ones. However, if green ABS is issued with external certification, green certifiers will monitor products’ environmental performance over the life cycle, and reputable intermediaries will annually review operating status with stricter standards than non-reputable ones. This mechanism incentivizes managers to exert high effort in operating projects and avoid conducting greenwashing behavior after green ABS issuances, thus mitigating ex-post moral hazard.

Previous studies on the value of green and intermediary certification are mainly focused on the stock and bond market. While several empirical papers have found no significant pricing effect of external reviews [11, 51], most research supports the presence of certification effects. For instance, Sundarasen et al. [52] state that auditors’ reputations increase demand in the secondary equity market, and Fang [8] demonstrates that the investment banks’ reputations reduce the offering yield of bonds. Similarly, Lyu et al. [17] reach consistent conclusions using data from the Chinese corporate bonds market. However, these studies only consider the certification effects of individual intermediaries. Moreover, Li et al. [53] and Simeth [4] find that green bonds with a certified green label have lower interest costs compared to those without them. Building upon these potential mechanisms and relevant empirical evidence, we propose Hypothesis 1 regarding the effect of external certification on the greenium of green ABS as follows:

H1. External certification has a negative impact on the greenium of green ABS.

### 2.3 Moderating effects of credit risk and information environment

Faltin-Traeger et al. [54] find a positive association between issuer credit quality and the performance of ABS products. In our context, low-quality green ABS issuers may greenwash intentionally instead of investing the proceeds in green projects. We then examine the interaction effects between issuer credit quality and the behavior of third parties and whether the former impacts the independence and effectiveness of the two external certifications.

Risky issuers may be associated with a higher risk of adverse selection. Specifically, these issuers are more likely to request green certifiers to conceal their greenwashing behaviors or collude with financial intermediaries to manipulate earnings. As external certification plays a signaling role only when the certificate is reliable, the certification cost needs to be sufficiently high to prevent low-quality issuers from easily adopting it. In comparison to the green certification market, financial intermediary markets are relatively mature, making intermediaries reject fraudulent activities due to the risk of damaging their reputation and future revenues [55, 56]. Moreover, the China Securities Regulatory Commission (CSRC) and the National Development and Reform Commission (NDRC) provide Chinese underwriters and credit rating agencies with official credit scores annually. These scores are endorsed by the strong government and valued by investors, potential issuers, and intermediaries, resulting in a lower likelihood of collusion between intermediaries and issuers. In contrast, the regulation policies in the green certification market are relatively lenient. After trading off the costs and benefits, low-quality issuers are more likely to collude with green certifiers instead of financial intermediaries. Thus, the signal provided by intermediary certification is more credible, and then pricing effects are more potent for ABS certified by reputable financial intermediaries.

The level of moral hazard of low-quality issuers may also be higher since they are more inclined to use the financing proceeds for non-green projects after issuance. Therefore, green certifiers and financial intermediaries monitor the environmental performance of these issuers more carefully and strictly throughout the entire life cycle, motivating them to improve their environmental performance. Consequently, both certifications are more effective in mitigating greenwashing risk for issuers with a higher credit risk. Based on the above analysis, we propose the following hypothesis:

**H2a.** External certification plays a signaling role, and the pricing effects of green and financial intermediary certification are, respectively, less and more significant for issuers with higher credit risk.
**H2b.** External certification plays an incentive role, and the pricing effects of green and financial intermediary certification are both more significant for issuers with higher credit risk.

Furthermore, the efficiency of conveying favorable information by external certification can also be influenced by the level of information asymmetry between issuers and investors, as well as the characteristics of the certification itself. It becomes challenging for investors to access sufficient public information [9, 16] in environments with limited transparency. In this case, reputable financial intermediaries possess valuable informational advantages due to their expertise, experiences, and access to various information channels [57, 58]. Intermediary certification thus plays a signaling role by providing incremental information and reducing information asymmetry. However, the credibility of a positive signal may also be met with skepticism under less transparent environments, particularly for a new signal in which the experience-based trust has yet to be built [59]. Besides, Chinese green ABS issuers predominantly engage domestic green certifiers for certification (over 95% of our sample), primarily due to lower fees. Nevertheless, some Chinese green certifiers may lack the required expertise and experience to effectively identify greenwashing behaviors and accurately predict environmental performance under high asymmetric information. As a result, there is more significant uncertainty in the private information of these certifiers, potentially leading to more noise and reduced credibility in their green certificates.

Issuers’ information asymmetry may also impact the ex-post incentive effect of external certification. Given the difficulties and high costs associated with regular third-party monitoring in less transparent information environments, it is challenging for certifiers to gather detailed information about eco-friendly activities and detect greenwashing behaviors. Consequently, certification is more likely to mitigate ex-post moral hazard for more transparent issuers, leading investors to perceive certified green ABS sold by transparent issuers as less risky. Based on the aforementioned analyses, we propose H3a and H3b as follows:

**H3a.** External certification plays a signaling role, and the pricing effects of green and financial intermediary certification are, respectively, less and more significant for less transparent issuers.
**H3b.** External certification plays an incentive role, and the pricing effects of green and financial intermediary certification are both less significant for less transparent issuers.

## 3. Data and model specification

### 3.1 Sample selection

The data used in this study are from 2016 to 2022, depending on the availability of green ABS market data in China. In total, we have 588 green ABS and 18,235 conventional ABS for our primary market analysis. During the matched set creation phase, we finally select 881 vanilla securities based on a range of restrictions. Our data collection primarily focuses on gathering information related to green certification, financial intermediary certification, and other characteristics of securities and issuers from the Wind database. The Wind database, a widely used data provider for Chinese ABS markets, contains valuable information on specific intermediaries involved in ABS issuance, such as third-party green certification agencies and financial institutions. However, due to the predominantly private placement nature of green ABS issuances, some data may be missing. In such cases, we manually obtain these missing values from the green ABS prospectuses. Additionally, data on rating agency reputation are hand-collected from the NDRC website, which provides credit scores of rating agencies and investment banks.

### 3.2 Variable definition

#### 3.2.1 Green ABS greenium

We adopt the methodological approach used by Zerbib [39] and match each green ABS based on several bond and issuer characteristics. We first calculate the ABS yield spread as the difference between the ABS offering yield and the corresponding Treasury bond spot yield with the same maturity [60]. Since interest rates change over time, this study matches the yield curve of Chinese Treasury securities based on the ABS’s issue date. Greeniums are then obtained by comparing conventional counterparts with the same issuer, debt rating, and similar bond maturity, size, issue year, and repayment sequence for each green ABS. In cases where the precise issuer requirements are too stringent, we relax the restriction for issuers in the same industry. Specifically, we narrow down eligible conventional ABS to those with an issue amount of RMB 0.1 billion more or less than the green ABS issue amount. We also limit the differences in issuance year and maturity to within two years and one year, respectively. As a result, we obtain 881 conventional ABS for 588 green ABS. Finally, the green premium *Greenium* in the green ABS offering yield spread is presented as follows:

$$Greenium_{i}=Sp_{i}^{G}-\frac{1}{N_{i}}\sum_{j=1}^{N_{i}}Sp_{j}^{C}$$ (1)

where $Sp_{i}^{G}$ stands the yield spread of the green ABS in match *i*, *N*_*i*_ represents the number of matches for the green ABS *i*, and $Sp_{1}^{C},\ldots ,Sp_{N_{i}}^{C}$ stand the yield spreads of the non-green ABS in match *i*.

#### 3.2.2 External certification

We utilize financial intermediary reputation as a proxy for financial intermediary certification. Since the issuance of green ABS involves four types of financial intermediaries (sponsors, underwriters, auditors, and rating agencies), this paper incorporates these intermediaries to construct the reputation index. The reputation variables are derived based on two principles: market share and official rating [8, 61]. According to the former principle, the reputations of sponsors, underwriters, and accounting firms are determined by their respective market shares in the ABS market, denoted as *Rps*, *Rpu*, and *Rpac*. As the market share of rating agencies is not available in the Wind database, we use the standardized credit scores assigned to credit rating agencies by NDRC as a proxy, denoted as *Rpr*.

Then, we conduct principal component analysis (PCA) to categorize the complex and inter-correlated set of reputation variables into a cohesive index. To obtain the combined reputation index *Rp*, PCA is performed on the standardized reputation variables *X*_*i*_. We set *b*_*ij*_ as the initial factor load, and the uncorrelated principal components *Z*_1_,*Z*_2_,…,*Z*_*m*_ are given by the linear transformation:

$$\begin{array}{l} Z_{1}=b_{11}X_{1}+b_{12}X_{2}+\ldots +b_{1m}X_{m}\\ Z_{2}=b_{21}X_{1}+b_{22}X_{2}+\ldots +b_{2m}X_{m}\\ \ldots \\ Z_{m}=b_{m1}X_{1}+b_{m2}X_{2}+\ldots +b_{mm}X_{m} \end{array}$$ (2)

The sample covariance matrix of **X** is $\hat{\Sigma }$; then, the eigenvectors *b*_1_,*b*_2_,…,*b*_*m*_ are obtained as follows:

Var[Zi]=argmaxVar[bi'X]s.t.{bi'bi=1bi'Σ^bj=0,fori≠j (3)

The corresponding eigenvalue are *λ*_1_,*λ*_2_,…,*λ*_*m*_, and the proportion of total variability explained by the first *k* principal components is:

$$p_{k}=\frac{\lambda_{1}+\ldots +\lambda_{k}}{\lambda_{1}+\ldots +\lambda_{m}}$$ (4)

Detailed information regarding the PCA results for the financial intermediary reputation is shown in **Table 1**. The first three principal components account for 44.01%, 25.06%, and 21.63% of the variations, respectively, with corresponding eigenvalues of 1.7603, 1.0024, and 0.8653. Since the cumulative explanatory power of the first three principal components reaches 90.70%, surpassing the threshold of 85%, we calculate an eigenvalue-weighted average reputation index based on them.

**Table 1:** Principal component analysis.

	Eigenvalue	Difference	Proportion	Cumulative
Component 1	1.7603	0.7579	44.01%	44.01%
Component 2	1.0024	0.1371	25.06%	69.07%
Component 3	0.8653	0.4933	21.63%	90.70%
Component 4	0.3720		9.3%	100%

Notes: This table shows the detailed information about principal component analysis results of financial intermediary reputation. Specifically, the eigenvalues of principal components, the differences between adjacent eigenvalues, the proportions and the cumulative proportions of principal components are reported.

Green certification serves as an explicit form of external certification. Some issuers attach third-party certification labels to green ABS after a rigorous assessment process [13]. We classify ABS as green-certified if it discloses a specific green certifier, drawing on the methodology outlined by Hu et al. [62]. Hence, we define the variable *Gre* as a binary indicator, taking a value of 1 if the green ABS is certified by a third-party green certification agency and zero otherwise.

#### 3.2.3 Moderator variables and other control variables

To examine the influence of external certification on the greenium of green ABS across different levels of issuer credit risk and information asymmetry, this paper incorporates issuer credit quality (*IRank*) and issuer age (*Dage*) as moderator variables in the empirical model. The model also includes several control variables that capture various green ABS and issuer characteristics. Regarding green ABS bond-specific characteristics, we control for the number of years to maturity (*Term*), the tranche credit rating of the green ABS (*BRank*), the size of the green ABS (*BSize*), the subordination level (*Sub*), and the frequency of interest payments (*Freq*). For issuer-specific characteristics, we consider control variables such as issuer size (*ISize*), profitability (*ROE*), leverage ratio (*Lev*), state-ownership (*SOE*), credit quality of the originators (*IRank*), firm age (*Dage*), and the level of operating cash flows (*CFO*). To mitigate the impact of outliers, we winsorize these control variables at the 1 and 99 percentiles. The detailed definitions of variables are presented in **Table 2**.

**Table 2:** Variable definition.

Variables	Definition
**Dependent variables**	
*Greenium*	Green ABS spread–the average of matching ABS spreads
**Independent variables**	
*Rp*	Determined by PCA based on four types of intermediary reputation variables, i.e., *Rps*, *Rpu*, *Rpac*, and *Rpr*
*Gre*	Equals 1 if the green ABS is certified as green and 0 otherwise
**Control variables**	
*Term*	The actual term of the green ABS (year)
*BRank*	Tranche credit rating: from 0 (A+ rated) to 3 (AAA rated)
*BSize*	The natural logarithm of green ABS issuance size (RMB 1 million)
*Sub*	The subordination level in green ABS
*Freq*	The number of coupon payments per year
*ISize*	The natural logarithm of the year-end value of total assets in the year before the ABS issue (RMB 1 million)
*ROE*	Net income divided by total assets for the year before the ABS issue
*Lev*	The ratio of total assets divided by total equity.
*SOE*	Takes a value of 1 if the issuer is state-owned, and 0 otherwise
*IRank*	Takes a value of 1 if the issuer has a rating, and 0 otherwise
*Dage*	Takes a value of 1 if the issuer’s age is larger than sample median, and 0 otherwise
*CFO*	Net operating cash flows divided by operating income.
**Moderator variables**	
*IRank*	Takes a value of 1 if the issuer has a rating, and 0 otherwise
*Dage*	Takes a value of 1 if the issuer’s age is larger than sample median, and 0 otherwise

Notes: This table presents definitions for the main variables. The reputations of sponsors, underwriters, and accounting firms are their corresponding market shares in the ABS market, labeled *Rps*, *Rpu*, and *Rpac*, respectively. We use the standardized credit scores of credit rating agencies by the National Development and Reform Commission (NDRC) as a proxy, labeled *Rpr*.

### 3.3 Model estimation technique

To explore the impact of external certification on green ABS greenium, we specify the following fixed-effect model:

$$\begin{array}{l} Greenium_{i}=\alpha_{0}+\alpha_{1}ExCertif_{i}+\sum_{k}\alpha_{k}bControl_{k,i}+\sum_{l}\alpha_{l}iControl_{l,i}\\ \;+\sum Industry+\sum Year+\varepsilon_{i} \end{array}$$ (5)

where the dependent variable *Greenium*_*i*_ is the greenium of the *i*th green ABS, and the core explanatory variable *ExCertif*_*i*_ is either *Rp*_*i*_ or *Gre*_*i*_ of the *i*th green ABS. *bControl*_*k*,*i*_ and *iControl*_*l*,*i*_ represent the vector of control variables, controlling for the green ABS bond- and issuer-specific characteristics, respectively. The error term is *ε*_*i*_. Industry and year fixed effects are also included. All standard errors are corrected for heteroscedasticity.

We further build a model to test the moderating effects of external certification in green ABS issuance. *Mod*_*i*_ represents *IRank*_*i*_ and *Dage*_*i*_ of the *i*th green ABS. We control for industry and year fixed effects. Specifically, the regression is constructed as follows:

$$\begin{array}{l} Greenium_{i}=\lambda_{0}+\lambda_{1}ExCertif_{i}+\lambda_{2}Mod_{i}+\lambda_{3}ExCertif_{i}\times Mod_{i}\\ \;+\sum_{k}\lambda_{k}bControl_{k,i}+\sum_{l}\lambda_{l}iControl_{l,i}+\sum Industry+\sum Year+\mu_{i} \end{array}$$ (6)

## 4. Empirical analysis

### 4.1 Descriptive statistics

**Table 3** displays the descriptive statistics for the variables used in our empirical study, providing valuable preliminary insights before conducting the regression analysis. Notably, the mean (−0.1317) and median (−0.0944) of the *Greenium* exhibit significantly negative values, indicating that the “greenness” factor can benefit issuers by reducing their financing costs as investors demonstrate pro-environmental preferences [39]. Interestingly, the sample median of financial intermediary reputation *Rp* (−0.1526) is lower than the sample mean (0.0007). Additionally, we observe a positive skewness (1.2258) in the distribution of intermediary reputation. This suggests that while some issuers engage prestigious financial intermediaries, a larger number of issuers opt for more affordable but non-reputable institutions. Moreover, about 30% of the green ABS in the sample are not certified by third-party green certification agencies.

**Table 3:** Descriptive statistics.

Variables	Mean	S.D.	Min.	p25	p50	p75	Max.	N
*Greenium*	−0.1317	1.0009	−3.1425	−0.7172	−0.0944	0.4266	3.2389	588
*Rp*	0.0007	0.7354	−0.9353	−0.5339	−0.1526	0.3964	2.9618	588
*Gre*	0.7160	0.4513	0	0	1	1	1	588
*Term*	4.0035	3.5139	0.2411	1.6589	2.9480	5.0438	17.6877	588
*BRank*	2.8214	0.4412	1	3	3	3	3	588
*BSize*	5.0958	1.4298	2.3026	3.9890	5.0056	6.1696	9.0108	588
*Sub*	5.9437	4.7802	0	4.9800	5.0500	5.8800	27.6000	588
*Freq*	3.2143	2.2723	0	2	4	4	120	588
*ISize*	9.1267	2.5225	3.2129	7.4491	9.1089	10.4582	16.5993	582
*ROE*	3.3961	13.5385	−49.6833	0.0609	4.6439	10.9225	36.2704	569
*Lev*	6.8163	6.5567	1.0930	2.9779	4.5470	7.5569	34.2087	573
*SOE*	0.8452	0.3620	0	1	1	1	1	588
*IRank*	0.4303	0.4955	0	0	0	1	1	588
*Dage*	0.4796	0.5000	0	0	0	1	1	588
*CFO*	−319.8514	2646.9242	−22400	−0.6725	0.1783	0.5483	3.0424	564

Notes: This table shows descriptive statistics for the main variables employed in the analysis.

### 4.2 Baseline regression

**Table 4** presents the regression results of Eq (5), which explores the value of external certification in green ABS issuance. Ordinary least squares (OLS) estimations are conducted with heteroskedasticity robust standard errors while controlling for year and industry fixed effects. Across all three columns, the coefficients of *Rp* and *Gre* are consistently and significantly negative at the 5% level regardless of controlling bond and issuer characteristics or not, indicating that both external certifications reduce the greenium of green ABS. That is, external certification effectively mitigates investors’ greenwashing concerns (i.e., skeptical voices questioning whether green ABS improve environmental performance or are just another case of greenwashing), which aligns to some extent with the findings of Simeth [4] and Yang et al. [63]. Thus, our findings support H1.

**Table 4:** Results on the impacts of external certification on the greenium of green ABS.

	(1)	(2)	(3)
Variables	*Greenium*	*Greenium*	*Greenium*
*Rp*	−0.1800***	−0.1782***	−0.1547**
	(−3.0871)	(−2.8581)	(−2.1975)
*Gre*	−0.4848***	−0.4675***	−0.2698**
	(−5.2546)	(−5.0278)	(−2.5246)
*Term*		0.0054	0.0042
		(0.4118)	(0.2817)
*BRank*		−0.0019	−0.0181
		(−0.0179)	(−0.1598)
*BSize*		−0.0347	−0.0359
		(−1.1094)	(−0.8463)
*Sub*		0.0234***	0.0229***
		(2.8892)	(2.6893)
*Freq*		0.0153	0.0240
		(0.9010)	(1.0598)
*ISize*			0.0547*
			(1.7966)
*ROE*			−0.0031
			(−0.7144)
*Lev*			0.0106
			(1.4820)
*SOE*			−0.0206
			(−0.1519)
*iRank*			−0.0036
			(−0.0296)
*Dage*			−0.5565***
			(−5.6196)
*CFO*			0.0000
			(1.1991)
Year FE	YES	YES	YES
Industry FE	YES	YES	YES
Constant	−0.6782***	−0.7306*	−0.9705**
	(−3.5660)	(−1.8614)	(−2.0259)
Observations	588	588	554
Adjusted *R*^*2*^	0.1419	0.1507	0.1727

Notes: This table shows the impact of external certification on the greenium of green ABS using the OLS approach. All variables are defined in **Table 2**. All models include year and industry fixed effects. *t* statistics in parentheses are robust to heteroskedasticity.

*** *p* < 0.01

** *p* < 0.05

* *p* <0.1.

Furthermore, our results are also economically significant. Based on column (3), one standard deviation (0.7354) increase in *Rp* is associated with a substantial 0.1138 decrease in the greenium of green ABS. Certifying green ABS as green is associated with a 0.2698 decrease in the green premium, accounting for 27% of its standard deviation (1.0001). In this context, issuers can save financing costs of 403 and 170 thousand RMB in greenium annually from green certification and financial intermediary certification (increasing *Rp* by one standard deviation), respectively, for green ABS where the size of the issue is about 149 million RMB (the sample median).

The model in our study includes several control variables to account for various bond and issuer characteristics. Among the control variables related to bond characteristics, we find that the higher level of subordination is associated with higher greenium, indicating that these ABS might have a higher risk of greenwashing. As for the control variables related to issuer characteristics, our findings suggest that older firms can issue green ABS at a lower greenium. Investors are more willing to pay higher ABS prices from mature issuers than from young issuers, because the level of information asymmetry and the perceived greenwashing risk of the former are lower. This finding aligns partially with the findings of Lyu et al. [17].

### 4.3 Endogeneity issue

To address self-selection bias, we employ the propensity score matching (PSM) method. This method helps mitigate the issue of certification effects potentially being driven by factors that predict external certification, such as financial conditions, rather than the certification itself. In order to estimate the propensity score, we utilize a logistic regression model with a binary dependent variable. However, one of our main explanatory variables, financial intermediary reputation (*Rp*), needs to be processed. We create a dummy variable (*Rp_D*) based on intermediary reputation. Specifically, we divide the sample into high- and low-reputation groups using the sample median of *Rp* as the threshold. We then match the green ABS externally certified by third parties against the green ABS not certified with different matching techniques, i.e., radius, kernel, and nearest neighbor matching.

The results in **Table 5** reveal that the coefficients of both green certification (*Gre*) and financial intermediary certification (*Rp_D*) are negative and significant at the 5% level regardless of the matching method. This indicates the presence of a pricing effect of external certification in terms of greenness even after accounting for endogenous matching through the propensity score method.

**Table 5:** Results on the role of external certification in green ABS issuance with the PSM method.

	Radius Matching (0.01)	Kernel Matching	1:5 K−nearest Matching	Radius Matching (0.01)	Kernel Matching	1:5 K−nearest Matching
	(1)	(2)	(3)	(4)	(5)	(6)
Variables	*Greenium*	*Greenium*	*Greenium*	*Greenium*	*Greenium*	*Greenium*
*Rp_D*	−0.1935**	−0.2030**	−0.2076**			
	(−2.0959)	(−2.1997)	(−2.1339)			
*Gre*				−0.3223***	−0.3436***	−0.3436***
				(−2.7133)	(−2.9091)	(−2.9091)
*Term*	−0.0006	−0.0031	0.0062	−0.0034	−0.0052	−0.0052
	(−0.0313)	(−0.1700)	(0.3448)	(−0.1816)	(−0.2767)	(−0.2767)
*BRank*	−0.1683	−0.2323	−0.2100	−0.0551	−0.0862	−0.0862
	(−1.0886)	(−1.5354)	(−1.3625)	(−0.4426)	(−0.6930)	(−0.6930)
*BSize*	−0.0598	−0.0720	−0.0613	−0.0463	−0.0452	−0.0452
	(−1.1310)	(−1.3945)	(−1.1287)	(−0.9791)	(−0.9566)	(−0.9566)
*Sub*	0.0206**	0.0193**	0.0197**	0.0307***	0.0257***	0.0257***
	(2.3276)	(2.2240)	(2.1908)	(3.9254)	(2.9768)	(2.9768)
*Freq*	−0.0251	−0.0279	−0.0140	0.0074	0.0077	0.0077
	(−0.7446)	(−0.8609)	(−0.4272)	(0.3317)	(0.3504)	(0.3504)
*ISize*	0.0602*	0.0621*	0.0730**	0.0617*	0.0602*	0.0602*
	(1.7155)	(1.8196)	(2.0619)	(1.8788)	(1.8494)	(1.8494)
*ROE*	−0.0109**	−0.0105**	−0.0128**	−0.0151***	−0.0130***	−0.0130***
	(−2.1677)	(−2.1316)	(−2.3789)	(−2.9447)	(−2.6777)	(−2.6777)
*Lev*	0.0225**	0.0253**	0.0236**	0.0103	0.0073	0.0073
	(2.1087)	(2.4147)	(2.0426)	(1.1132)	(0.8088)	(0.8088)
*SOE*	−0.0652	−0.0268	−0.0231	−0.0015	0.0234	0.0234
	(−0.4094)	(−0.1700)	(−0.1428)	(−0.0102)	(0.1588)	(0.1588)
*iRank*	0.0304	0.0608	−0.0292	0.0737	0.0602	0.0602
	(0.2150)	(0.4536)	(−0.2098)	(0.5433)	(0.4458)	(0.4458)
*Dage*	−0.6469***	−0.6559***	−0.6745***	−0.6689***	−0.6398***	−0.6398***
	(−4.7743)	(−4.8528)	(−4.9327)	(−5.4673)	(−5.1497)	(−5.1497)
*CFO*	0.0159	0.0161	0.0145	0.0000**	0.0000**	0.0000**
	(1.3384)	(1.3644)	(1.2549)	(2.5398)	(2.5158)	(2.5158)
Constant	−0.4347	−0.1724	−0.3780	−0.5571	−0.4486	−0.4486
	(−0.7670)	(−0.3125)	(−0.6828)	(−1.1649)	(−0.9359)	(−0.9359)
Year FE	YES	YES	YES	YES	YES	YES
Industry FE	YES	YES	YES	YES	YES	YES
Observations	470	484	434	505	509	509
Adjusted *R*^*2*^	0.1615	0.1577	0.1594	0.1559	0.1460	0.1460

Notes: This table alleviate the endogeneity concerns of our baseline findings by using the propensity score matching (PSM) approach. We consider radius, kernel, and nearest neighbor matching. Before using a logistic regression model to estimate the propensity score, we construct a dummy variable *Rp_D*, which equals 1 if the financial intermediary reputation is above the sample median and 0 otherwise. All the other variables are defined in **Table 2**. All models include year and industry fixed effects. *t* statistics in parentheses are robust to heteroskedasticity.

*** *p* < 0.01

** *p* < 0.05

* *p* <0.1.

### 4.4 Additional robustness tests

In this section, we conduct three sets of additional robustness checks on H1. The first robustness issue is that the previous results may hinge on the specific calculation method of financial intermediary reputation. Consequently, we replace the basic reputation variables with dummy variables. Specifically, we assign a value of 1 to auditors belonging to international big four auditing firms (i.e., PricewaterhouseCoopers (PwC), Deloitte, Ernst & Young (EY), and Klynveld Peat Marwick Goerdeler (KPMG)) or domestic big four firms (i.e., ShuLun Pan, Ruihua, Pan-China, and Da Hua) and distinguish other financial intermediaries into reputable and non-reputable groups based on their sample medians. Then, we construct alternative explanatory variables using PCA and re-estimate the regressions. The results in **Table 6** show that the coefficients of *DRp*_*pca*_ and *Gre* remain negative and significant at the 5% level, indicating that the impacts of external certification on the greenium of green ABS are still statistically and economically significant.

**Table 6:** Results of robustness test using alternative *Rp* measures.

	(1)	(2)	(3)
Variables	*Greenium*	*Greenium*	*Greenium*
*Rp*	−0.3627***	−0.3553***	−0.3751***
	(−5.5509)	(−4.8433)	(−4.4718)
*Gre*	−0.4065***	−0.4198***	−0.2489**
	(−4.3435)	(−4.4538)	(−2.3520)
*Term*		−0.0010	0.0006
		(−0.0763)	(0.0437)
*BRank*		−0.0176	−0.0135
		(−0.1693)	(−0.1216)
*BSize*		0.0085	0.0088
		(0.2540)	(0.2039)
*Sub*		0.0134*	0.0120
		(1.6619)	(1.3638)
*Freq*		0.0176	0.0269
		(1.0899)	(1.2364)
*ISize*			0.0635**
			(2.1435)
*ROE*			−0.0007
			(−0.1729)
*Lev*			0.0089
			(1.2836)
*SOE*			−0.0584
			(−0.4313)
*iRank*			−0.1003
			(−0.8255)
*Dage*			−0.4693***
			(−4.7356)
*CFO*			0.0000
			(1.3747)
Year FE	YES	YES	YES
Industry FE	YES	YES	YES
Constant	−0.7942***	−0.9134**	−1.3953***
	(−3.7446)	(−2.2618)	(−2.7703)
Observations	588	588	554
Adjusted *R*^*2*^	0.1791	0.1782	0.2008

Notes: This table uses alternative measures of the financial intermediary reputation to check the reliability of our main results. We first distinguish the sponsors, the lead underwriters and the credit rating agencies into reputable and non-reputable groups based on sample medians. Besides, well-known auditors are defined as international or domestic big four accounting firms (i.e., PricewaterhouseCoopers (PwC), Deloitte, Ernst & Young (EY), Klynveld Peat Marwick Goerdeler (KPMG), ShuLun Pan, Ruihua, Pan-China, and Da Hua.). Then we construct alternative explanatory variables using PCA, labeled *DRp_pca_*. All the other variables are defined in **Table 2**. All models include year and industry fixed effects. *t* statistics in parentheses and are robust to heteroskedasticity.

*** *p* < 0.01

** *p* < 0.05

* *p* <0.1.

Secondly, we replace the explained variables *Greenium*. Fang [8] examines the certification effect of financial intermediary reputation using offering yield rather than treasury yield spread. Thus, we here measure the greenium by the difference in offering yield between green and comparable non-green ABS. As shown in **Table 7**, externally certified ABS are subject to a lower greenium while the coefficient on *Rp* seems to be statistically insignificant once issuer characteristics have been controlled. Finally, **Table 8** includes industry-year fixed effects to control for all time-varying industry-specific characteristics. Again, the results in **Table 8** are qualitatively similar to the baseline findings. Overall, H1 is supported robustly.

**Table 7:** Results of robustness test using alternative *Greenium* measures.

	(1)	(2)	(3)
Variables	*Greenium*	*Greenium*	*Greenium*
*Rp*	−0.1516**	−0.1592**	−0.1088
	(−2.2107)	(−2.2034)	(−1.3624)
*Gre*	−0.5045***	−0.4763***	−0.2110*
	(−5.0088)	(−4.6919)	(−1.8697)
*Term*		0.0075	0.0020
		(0.6176)	(0.1401)
*BRank*		0.0863	0.0285
		(0.7863)	(0.2500)
*BSize*		−0.0560*	−0.0231
		(−1.7089)	(−0.5210)
*Sub*		0.0270***	0.0284***
		(2.6994)	(2.8559)
*Freq*		0.0318*	0.0428*
		(1.6603)	(1.7985)
*ISize*			0.0317
			(1.0149)
*ROE*			−0.0078*
			(−1.8915)
*Lev*			0.0113*
			(1.6880)
*SOE*			0.0709
			(0.4984)
*iRank*			−0.0009
			(−0.0076)
*Dage*			−0.6481***
			(−6.0702)
*CFO*			0.0000
			(1.3505)
Year FE	YES	YES	YES
Industry FE	YES	YES	YES
Constant	−1.1733***	−1.4726***	−1.5146***
	(−5.3873)	(−3.4520)	(−2.9205)
Observations	588	588	554
Adjusted *R*^*2*^	0.1913	0.2057	0.2394

Notes: This table uses alternative measures of the greenium to check the reliability of our main results. We calculate the difference in yield between green and non-green ABS to proxy for the greenium. All the other variables are defined in **Table 2**. All models include year and industry fixed effects. *t* statistics in parentheses and are robust to heteroskedasticity. *** *p* < 0.01

** *p* < 0.05

* *p* <0.1.

**Table 8:** Results of robustness test including other dimensional fixed effects.

	(1)	(2)	(3)
Variables	*Greenium*	*Greenium*	*Greenium*
*Rp*	−0.2265***	−0.2106***	−0.2079***
	(−3.8935)	(−3.3385)	(−2.9033)
*Gre*	−0.4459***	−0.4304***	−0.2032*
	(−4.3930)	(−4.1976)	(−1.7714)
*Term*		0.0085	0.0077
		(0.6338)	(0.5155)
*BRank*		−0.0454	−0.0198
		(−0.4327)	(−0.1788)
*BSize*		−0.0601	−0.0535
		(−1.6434)	(−1.2320)
*Sub*		0.0109	0.0052
		(1.2224)	(0.5253)
*Freq*		0.0236	0.0203
		(1.3356)	(0.9069)
*ISize*			0.0522*
			(1.6974)
*ROE*			−0.0075
			(−1.4497)
*Lev*			0.0048
			(0.6341)
*SOE*			−0.2766*
			(−1.6845)
*iRank*			−0.1640
			(−1.1847)
*Dage*			−0.4721***
			(−4.2833)
*CFO*			−0.0000
			(−1.3045)
Year FE	YES	YES	YES
Industry FE	YES	YES	YES
Year* Industry FE	YES	YES	YES
Constant	−1.0581***	−0.7836*	−0.7461
	(−5.0475)	(−1.8211)	(−1.4336)
Observations	588	588	554
Adjusted *R*^*2*^	0.1761	0.1814	0.2037

Notes: This table uses industry-year fixed effects to control for all time-varying industry-specific characteristics and check the reliability of our main results. All variables are defined in **Table 2**. All models include year and industry fixed effects. *t* statistics in parentheses and are robust to heteroskedasticity.

*** *p* < 0.01

** *p* < 0.05

* *p* <0.1.

### 4.5 Mechanism analyses

#### 4.5.1 Moderating effect of issuer credit risks

In this section, we explore whether the effects of green certificates and client relationships with prestigious financial intermediaries are associated with green ABS issuer’s quality by selecting the issuer’s credit rating (*IRank*) as the moderating variable. We then introduce its interactions with *Rp* and *Gre*, namely *IRank×Rp* and *IRank×Gre*, and estimate Eq (6).

**Table 9** reports the results on financial intermediary certification in columns (1)−(3), while columns (4)−(6) report the results on green certification. As shown in the first three columns, *IRank×Rp* has a significant positive impact on *Greenium*, with regression coefficients slightly decreased from 0.37 to 0.22 after accounting for a range of control variables. These coefficients indicate that the certification effect of intermediary reputation is stronger for an issuing firm with higher credit risk. Conversely, the coefficient of *IRank×Gre* is −0.34 when we control for issuer and bond characteristics, suggesting that the pricing effect of green certificate is weaker for issuing firms with lower credit quality. Therefore, our findings support H2a; that is, external certification plays a signaling role in green ABS issuance.

**Table 9:** Results of the moderating effect of the issuer’s credit risk.

	(1)	(2)	(3)	(4)	(5)	(6)
Variables	*Greenium*	*Greenium*	*Greenium*	*Greenium*	*Greenium*	*Greenium*
*IRank×Rp*	0.3700***	0.3522***	0.2201*			
	(3.2830)	(3.0583)	(1.6770)			
*IRank×Gre*				−0.0295	−0.0415	−0.3376*
				(−0.1656)	(−0.2313)	(−1.8454)
*Rp*	−0.4657***	−0.4491***	−0.2975***			
	(−5.4800)	(−4.9663)	(−2.9634)			
*Gre*				−0.5427***	−0.5061***	−0.1358
				(−4.7611)	(−4.3261)	(−1.0311)
*iRank*	−0.0995	−0.0886	−0.0150	−0.0679	−0.0296	0.2729
	(−1.1965)	(−0.9933)	(−0.1254)	(−0.4383)	(−0.1842)	(1.5070)
*Term*		0.0112	0.0087		0.0039	0.0022
		(0.9462)	(0.6651)		(0.3352)	(0.1734)
*BRank*		0.0472	0.0037		−0.0161	−0.0003
		(0.4844)	(0.0363)		(−0.1649)	(−0.0032)
*BSize*		−0.0286	−0.0411		−0.0504	−0.0657
		(−0.8148)	(−0.9949)		(−1.4909)	(−1.6133)
*Sub*		0.0271***	0.0257***		0.0251***	0.0245***
		(2.9293)	(2.7341)		(2.7310)	(2.6126)
*Freq*		0.0046	0.0143		−0.0025	0.0065
		(0.2277)	(0.6144)		(−0.1269)	(0.2927)
*ISize*			0.0639**			0.0557*
			(2.2168)			(1.9142)
*ROE*			−0.0065*			−0.0048
			(−1.6865)			(−1.2321)
*Lev*			0.0091			0.0107
			(1.2396)			(1.5101)
*SOE*			0.0473			−0.0242
			(0.3349)			(−0.1757)
*Dage*			−0.6124***			−0.6253***
			(−5.4008)			(−5.3987)
*CFO*			0.0000			0.0000
			(1.0549)			(1.4481)
Constant	−0.9462***	−1.1788***	−1.2326***	−0.5079**	−0.4226	−0.7540
	(−4.5328)	(−3.0749)	(−2.6101)	(−2.2803)	(−1.1058)	(−1.6259)
Year FE	YES	YES	YES	YES	YES	YES
Industry FE	YES	YES	YES	YES	YES	YES
Observations	588	588	554	588	588	554
Adjusted *R*^*2*^	0.1165	0.1256	0.1660	0.1276	0.1374	0.1697

Notes: All variables are defined in **Table 2**. Year and industry fixed effects are controlled. *t* statistics are reported in parentheses and are robust to heteroskedasticity.

*** *p* < 0.01

** *p* < 0.05

* *p* <0.1. These notes also apply to **Tables 10**–12.

#### 4.5.2 Moderating effect of information environment

We then shift our focus to the information environment of green ABS issuers and investigate whether it influences the external certification effect. We introduce *Dage* as a moderating variable and estimate the Eq (6). **Table 10** reports the results from the regression without and with control variables included. The coefficients of *Dage×Rp* are displayed in columns (1)−(3), which are 0.4240, 0.3992, and 0.3450, respectively. These interaction effects are significant at the 5% level, suggesting that issuers’ information asymmetry strengthens the pricing effect of intermediaries’ reputation. However, as shown in column (4)−(6), the coefficient of *Dage×Gre* is significantly negative at 5% level. This indicates that the green certificate is less effective for opaque issuing firms. Therefore, the endorsement from a reputation-oriented or independent third party serves as a positive signal of the product quality and H3a is supported.

**Table 10:** Results of the moderating effect of the issuer’s information environment.

	(1)	(2)	(3)	(4)	(5)	(6)
Variables	*Greenium*	*Greenium*	*Greenium*	*Greenium*	*Greenium*	*Greenium*
*Dage×Rp*	0.4240***	0.3992***	0.3450**			
	(3.8325)	(3.5371)	(2.4686)			
*Dage×Gre*				−0.5790***	−0.5215**	−0.6166***
				(−2.8683)	(−2.3983)	(−2.8109)
*Rp*	−0.4464***	−0.4213***	−0.3780***			
	(−6.1299)	(−5.5099)	(−3.8383)			
*Gre*				−0.2697**	−0.2673**	−0.0596
				(−2.5742)	(−2.4287)	(−0.4567)
*Dage*	−0.4834***	−0.4862***	−0.5973***	−0.0122	−0.0478	−0.1488
	(−5.8762)	(−5.5565)	(−5.9614)	(−0.0743)	(−0.2601)	(−0.8075)
*Term*		0.0062	0.0060		−0.0006	0.0013
		(0.5121)	(0.4061)		(−0.0488)	(0.0832)
*BRank*		−0.0533	−0.0205		−0.0447	0.0139
		(−0.4983)	(−0.1772)		(−0.4288)	(0.1211)
*BSize*		−0.0183	−0.0393		−0.0413	−0.0739*
		(−0.5511)	(−0.8724)		(−1.2473)	(−1.6884)
*Sub*		0.0249***	0.0222***		0.0213***	0.0192**
		(3.1465)	(2.6257)		(2.6108)	(2.1688)
*Freq*		0.0005	0.0130		0.0021	0.0104
		(0.0257)	(0.5795)		(0.1247)	(0.4860)
*ISize*			0.0461			0.0531
			(1.4500)			(1.6214)
*ROE*			−0.0052			−0.0040
			(−1.3335)			(−0.8652)
*Lev*			0.0126*			0.0126*
			(1.8123)			(1.8038)
*SOE*			0.0120			−0.0238
			(0.0870)			(−0.1717)
*iRank*			0.0600			0.0115
			(0.4808)			(0.0906)
*CFO*			−0.0000			0.0000***
			(−0.1379)			(2.6180)
Constant	−0.5580***	−0.4920	−1.0052**	−0.3728**	−0.1823	−0.7662
	(−2.7667)	(−1.2609)	(−2.0558)	(−1.9877)	(−0.5016)	(−1.5970)
Year FE	YES	YES	YES	YES	YES	YES
Industry FE	YES	YES	YES	YES	YES	YES
Observations	588	588	554	588	588	554
Adjusted *R*^*2*^	0.1621	0.1699	0.1726	0.1742	0.1812	0.1794

### 4.6 Heterogeneity analyses

#### 4.6.1 Different issuer industries

This paper further conducts a series of heterogeneity tests in this section. We first examine whether there is heterogeneity in the impact of external certification on the greenium of green ABS among issuers among different industry types. As indicated by the previous analysis, external certification mitigates the greenwashing concern through the signaling channel. Given that financial institutions’ lending business relies on private information, the underlying assets of financial ABS issuers can lack transparency, leading to greater difficulty in green certification. Additionally, there is a higher probability of collusion between financial issuers and financial intermediaries because there are close contractual relationships among financial institutions. Consequently, the credibility of a positive signal conveyed to investors is more likely to be doubtful, and the pricing effect of the certification is expected to be weaker for financial institutions compared to non-financial firms [41].

We divide our sample into two groups: financial and other industries. As shown in **Table 11**, the coefficients of *Rp* (0.0180, 0.0400, and 0.0842) in the financial industry subsample are not statistically significant at the 10% level, whereas the coefficients of *Rp* (−0.3949, −0.3898, and −0.3750) in the non-financial industry subsample are statistically significant at the 1% level. These results suggest that the impact of intermediaries’ reputations on green ABS pricing of greenness is more pronounced for non-financial issuers, both in terms of statistical significance and economic magnitude. We also observe similar results when considering the effect of green certification. This finding supports our conjecture that the external certification effect is more substantial for green ABS issued by non-financial corporations.

**Table 11:** Results of heterogeneity analysis by industry.

	Financial industry			Non-financial industry		
	(1)	(2)	(3)	(4)	(5)	(6)
Variables	*Greenium*	*Greenium*	*Greenium*	*Greenium*	*Greenium*	*Greenium*
*Rp*	0.0180	0.0400	0.0842	−0.3949***	−0.3898***	−0.3750***
	(0.3215)	(0.6949)	(1.0004)	(−4.6181)	(−4.3159)	(−3.7414)
*Gre*	−0.2185**	−0.1759	−0.2044*	−0.5527***	−0.4916***	−0.1394
	(−2.1493)	(−1.5797)	(−1.7398)	(−4.5429)	(−3.8650)	(−0.8822)
*Term*		0.0182	0.0171		0.0127	0.0029
		(1.3691)	(0.7767)		(0.8137)	(0.1713)
*BRank*		−0.0648	−0.0976		0.1194	0.1639
		(−0.5076)	(−0.6811)		(0.8930)	(1.1822)
*BSize*		−0.0206	−0.0002		−0.0365	−0.0307
		(−0.4509)	(−0.0026)		(−0.8438)	(−0.5585)
*Sub*		−0.0148	−0.0163		0.0286**	0.0168
		(−1.3278)	(−1.1769)		(2.3601)	(1.1898)
*Freq*		−0.0108	0.0083		−0.0034	−0.0230
		(−0.7255)	(0.4494)		(−0.0949)	(−0.5971)
*ISize*			−0.0498			0.0250
			(−0.7386)			(0.5940)
*ROE*			−0.0059			−0.0087*
			(−0.6381)			(−1.8286)
*Lev*			0.0001			−0.0105
			(0.0014)			(−1.2366)
*SOE*			0.0711			−0.3876
			(0.4260)			(−1.4309)
*iRank*			0.2399			−0.2002
			(1.4496)			(−1.1675)
*Dage*			0.0026			−0.6141***
			(0.0144)			(−4.7436)
*CFO*			0.0000			0.0821**
			(0.8021)			(2.0446)
Constant	−0.4347	−0.1724	−0.3780	−0.5571	−0.4486	−0.4486
	(−0.7670)	(−0.3125)	(−0.6828)	(−1.1649)	(−0.9359)	(−0.9359)
Year FE	YES	YES	YES	YES	YES	YES
Industry FE	NO	NO	NO	YES	YES	YES
Observations	151	151	140	437	437	414
Adjusted *R*^*2*^	0.0654	0.0577	0.0067	0.2012	0.2042	0.2544

#### 4.6.2 Different bond markets

We conduct a separate analysis to investigate the influence of external certification on green ABS issuance in two distinct markets: the interbank market and the securities exchange market. The interbank market mainly comprises financial institutions as investors [64], and the composition of market participants remains relatively stable, fostering familiarity among members. Conversely, the exchange market exhibits a higher level of anonymity among investors and greater information asymmetry. In such a setting, investors in the exchange market rely more heavily on certification signals to assess the quality of green ABS products.

As shown in **Table 12**, it exerts a more substantial influence on lowering greenium in both statistical and economic terms for green ABS issued in the securities exchange market. Specifically, the coefficients of *Rp* and *Gre* are −0.2766 and −0.3517 after controlling bond and issuer characteristics, with the 1% significance level. Consequently, green ABS issuers seeking financing in the securities exchange market should emphasize external certification more.

**Table 12:** Results of heterogeneity analysis by bond markets.

	Interbank market			Securities exchange market		
	(1)	(2)	(3)	(4)	(5)	(6)
Variables	*Greenium*	*Greenium*	*Greenium*	*Greenium*	*Greenium*	*Greenium*
*Rp*	−0.0363	0.0283	0.1789	−0.2776***	−0.1895**	−0.2766***
	(−0.4214)	(0.2933)	(1.0303)	(−3.6389)	(−2.3217)	(−2.8283)
*Gre*	−0.0244	−0.0617	0.5989*	−0.5343***	−0.5203***	−0.3517***
	(−0.1200)	(−0.2424)	(1.8444)	(−4.9443)	(−4.7701)	(−2.8566)
*Term*		0.0496	0.0726		0.0053	−0.0014
		(0.8201)	(1.3733)		(0.4185)	(−0.1015)
*BRank*		−0.0877	−0.1079		0.1088	0.0960
		(−0.4063)	(−0.4689)		(0.8772)	(0.7357)
*BSize*		0.0211	0.0766		−0.0554	0.0081
		(0.3136)	(0.5683)		(−1.4577)	(0.1607)
*Sub*		0.0031	0.0070		0.0498***	0.0415**
		(0.2490)	(0.4733)		(3.3133)	(2.2967)
*Freq*		−0.0447**	−0.0595*		0.0173	0.0178
		(−2.2037)	(−1.6984)		(0.5753)	(0.5474)
*ISize*			−0.0865			0.0004
			(−0.9983)			(0.0087)
*ROE*			−0.0388			−0.0019
			(−1.5335)			(−0.4249)
*Lev*			0.0750*			−0.0037
			(1.8690)			(−0.4967)
*SOE*			−0.5999**			−0.0153
			(−2.2729)			(−0.0954)
*iRank*			−0.0945			−0.0608
			(−0.4114)			(−0.3771)
*Dage*			−0.1670			−0.4761***
			(−0.3857)			(−4.4780)
*CFO*			0.0002			−0.0000
			(0.3297)			(−1.1816)
Constant	−0.3854*	−0.1441	1.1433	−0.7720***	−1.1243**	−1.2009**
	(−1.7260)	(−0.1896)	(0.6274)	(−3.3699)	(−2.2896)	(−1.9685)
Year FE	YES	YES	YES	YES	YES	YES
Industry FE	YES	YES	YES	YES	YES	YES
Observations	109	109	98	479	479	456
Adjusted *R*^*2*^	0.0037	0.0203	0.1813	0.1944	0.2057	0.2303

#### 4.6.3 Different types of financial intermediaries

In this section, we analyze the heterogeneity of certification effects among different types of financial intermediaries in green ABS issuance. Specifically, we focus on sponsors, lead underwriters, auditors, and credit rating agencies. The estimated results are displayed in **Table 13**. We notice that the reputations of accounting firms and credit rating agencies have minimal effect on green premiums, no matter whether control variables are included. Given that credit rating agencies and auditors mainly examine the cash flow, profitability, and solvency of the underlying assets, their reputation attests to the credit quality of green ABS and, therefore, affects the vanilla ABS spread component of the green ABS yield spread rather than the greenium.

**Table 13:** Results of heterogeneity analysis by financial intermediaries.

	(1)	(2)	(3)	(4)	(5)	(6)	(7)	(8)
Variables	*Greenium*	*Greenium*	*Greenium*	*Greenium*	*Greenium*	*Greenium*	*Greenium*	*Greenium*
*Rps*	−2.3338**				−1.0242			
	(−2.1323)				(−0.7823)			
*Rpu*		−7.1577***				−6.5117***		
		(−5.8007)				(−4.2939)		
*Rpac*			0.6699				1.1054	
			(1.1411)				(1.4045)	
*Rpr*				−0.0322				0.0861
				(−0.4329)				(1.0863)
*Gre*	−0.5105***	−0.4255***	−0.5635***	−0.5611***	−0.2869***	−0.2305**	−0.2801**	−0.2832***
	(−5.3987)	(−4.6735)	(−6.1793)	(−6.1526)	(−2.6445)	(−2.1436)	(−2.5449)	(−2.6552)
*Term*					0.0022	0.0067	0.0031	0.0044
					(0.1475)	(0.4516)	(0.2038)	(0.2884)
*BRank*					−0.0263	0.0031	−0.0256	−0.0274
					(−0.2348)	(0.0277)	(−0.2312)	(−0.2451)
*BSize*					−0.0468	−0.0302	−0.0583	−0.0571
					(−1.0674)	(−0.7290)	(−1.3662)	(−1.3416)
*Sub*					0.0239***	0.0157*	0.0232***	0.0252***
					(2.8032)	(1.8012)	(2.7438)	(2.9944)
*Freq*					0.0128	0.0358	0.0047	0.0084
					(0.5827)	(1.6388)	(0.2178)	(0.3936)
*ISize*					0.0520*	0.0480	0.0416	0.0466
					(1.6565)	(1.5821)	(1.2549)	(1.4352)
*ROE*					−0.0040	−0.0038	−0.0056	−0.0050
					(−0.9178)	(−0.8878)	(−1.2415)	(−1.1236)
*Lev*					0.0103	0.0088	0.0095	0.0105
					(1.4561)	(1.2491)	(1.3690)	(1.4768)
*SOE*					−0.0077	−0.0378	−0.0028	−0.0085
					(−0.0559)	(−0.2742)	(−0.0207)	(−0.0631)
*iRank*					0.0100	−0.0077	0.0348	0.0321
					(0.0793)	(−0.0630)	(0.2719)	(0.2484)
*Dage*					−0.5770***	−0.4920***	−0.5956***	−0.6143***
					(−5.7801)	(−5.0913)	(−6.0115)	(−6.3148)
*CFO*					0.0000*	−0.0000	0.0000**	0.0000***
					(1.8031)	(−0.4496)	(2.3450)	(2.8158)
Constant	−0.5523***	−0.5963***	−0.6068***	−0.5305***	−0.7066	−0.9688**	−0.5715	−0.6612
	(−3.0145)	(−3.2386)	(−3.3404)	(−2.7655)	(−1.4872)	(−2.0461)	(−1.2167)	(−1.4177)
Year FE	YES	YES	YES	YES	YES	YES	YES	YES
Industry FE	YES	YES	YES	YES	YES	YES	YES	YES
Observations	588	588	588	588	554	554	554	554
Adjusted *R*^*2*^	0.1344	0.1685	0.1283	0.1276	0.1654	0.1903	0.1662	0.1663

Notes: This table displays the differentiated certification effect of different types of financial intermediaries. The reputations of sponsors, underwriters, accounting firms, and credit rating are labeled *Rps*, *Rpu*, *Rpac*, and *Rpr*, respectively. All the other variables are defined in **Table 2**. All models include year and industry fixed effects. *t* statistics in parentheses are robust to heteroskedasticity.

*** *p* < 0.01

** *p* < 0.05

* *p* <0.1.

As for the lead underwriters and sponsors, their reputation has significant negative effects on the greenium of green ABS. That is, hiring prestigious sponsors and underwriters reduces the green ABS funding costs primarily by influencing the greenium. In China, where environmental information disclosure is insufficient, companies may engage in greenwashing practices to obtain lower financing costs. When reputable sponsors and underwriters are involved in self-labeled green ABS, they not only assess the financial quality of the projects but also identify and address greenwashing practices. Their involvement assures investors regarding the authenticity and credibility of the green attributes of green ABS, thus leading to a reduction in greenwashing concerns and the greenium. We conclude from this result that the certification effect of financial intermediaries is closely tied to the specific services they provide.

## 5. Conclusion and policy implications

Using Chinese green ABS issuance data from 2016 to 2022, we examine whether and how green and financial intermediary certification are reflected in the financing costs of green ABS. Our results suggest that both external certifications significantly reduce the greenium of green ABS by mitigating greenwashing concerns, especially for non-financial firms and ABS issued in the security exchange market. This pricing effect of both certifications stems from their signaling role in the pre-issuance period, instead of their incentive role during the post-issuance period in the green ABS market. Moreover, our analysis reveals differences between green and financial intermediary certification based on a moderating model. The results show that the credit risk and lack of transparency associated with green ABS issuers improve the signaling effect of financial intermediary certification but diminish that of green certification. That is, the signaling effect of certification depends on its credibility. Overall, our study confirms external certification as a valuable instrument for green ABS issuers who actually realize their environmental commitment to reduce financing barriers arising from information asymmetry.

Our results have some important policy implications and managerial insights. Firstly, policymakers, especially those in developing countries, should prioritize the gradual development of a robust and healthy green ABS market. By considering two different definitions of green securitization, green issuers, whose underlying asset pool is green, and non-green issuers, who invest the proceeds in green projects, can both effectively reduce financing barriers and further achieve green transition. Secondly, national governments should actively intervene to enhance the credibility and effectiveness of certification processes. This can be achieved by implementing standardized business processes for certifiers, enhancing specific supervision for their operations, or, like the Chinese government, regularly providing certifiers (financial intermediaries) with official credit scores. Finally, this paper offers guidance for different types of green ABS issuers in the choice of specific tools. Our empirical findings suggest that the effectiveness of certification depends on various factors, such as the issuer’s financial condition and information environment. Thus, potential issuers should strategically utilize external certification tools with careful consideration since the application and process of external certification can be costly. For example, young clean-energy technology companies with high opacity and low credit quality may benefit more from financial intermediary certification.

Certainly, our results have several limitations primarily owing to inadequate information disclosure requirements in the Chinese green ABS market. The severity of greenwashing concerns among investors may vary across different types of green ABS, ranging from those with green underlying assets to those with green use of proceeds. However, due to data availability restrictions, we are unable to further explore the differentiated effect of external certification on these two types of green ABS. Moreover, our empirical model does not account for factors such as the greenness and the concentration of underlying assets, as well as the correlation between them, all of which play critical roles in green ABS pricing.

This study can be further extended in several aspects. Firstly, future research can explore the differences in terms of greenium and pricing effect of external certification under green, brown, and even stranded underlying assets. However, this will most likely be achieved through a theoretical model or numerical analysis. Secondly, it will be significant and meaningful to investigate the motivations behind green ABS issuance. Thirdly, extending our study to a global level and then revealing differences among various countries could also be an interesting direction for future research.

## Supporting information

S1 Data (ZIP)
